# Temporal Trends in Cardiovascular Mortality in Underlying Viral Hepatitis: A Retrospective Analysis of Gender, Racial/Ethnic, and Regional Disparities

**DOI:** 10.1002/jgh3.70235

**Published:** 2025-07-27

**Authors:** Wania Sultan, Haider Ashfaq, Hamza Ashraf, Ahmad Khan, Ayman Omair Hashmi, Muhammad Omar Larik, Maheen Zahid, Yasir Majeed, Pratik Bhattarai, Ashujot K. Dang, Ahmed Ali Aziz, Hafiz Muhammad Sharjeel Arshad

**Affiliations:** ^1^ Department of Medicine Dow Medical College Karachi Pakistan; ^2^ Department of Medicine Allama Iqbal Medical College Lahore Pakistan; ^3^ Department of Medicine Khyber Medical University Peshawar Pakistan; ^4^ Department of Obstetrics and Gynecology Indus Hospital Karachi Pakistan; ^5^ Department of Medicine Dow International Medical College Karachi Pakistan; ^6^ Department of Medicine Liaquat University of Medical and Health Sciences Jamshoro Pakistan; ^7^ Department of Medicine King Edward Medical University Lahore Pakistan; ^8^ Department of Medicine Manipal College of Medical Sciences Pokhara Nepal; ^9^ Department of Medicine University of California Riverside School of Medicine Riverside California USA; ^10^ Department of Medicine Integris Baptist Medical Center Oklahoma City Oklahoma USA; ^11^ Division of Gastroenterology Southern Illinois University School of Medicine Springfield Illinois USA

**Keywords:** cardiovascular mortality, CDC‐WONDER, hepatitis B, hepatitis C, viral hepatitis

## Abstract

**Objective:**

This study aimed to investigate contemporary trends in mortality related to cardiovascular disease and viral hepatitis.

**Methods:**

We conducted a retrospective analysis using data from the CDC‐WONDER dataset. Our study cohort consisted of adults aged ≥ 15 years, where both cardiovascular disease and viral hepatitis were identified as an underlying or contributory cause of death between 1999 and 2020. Crude and age‐adjusted mortality rates (AAMR) per 1 000 000 population were extracted. Joinpoint regression analysis was utilized to calculate annual percentage change (APC) of each trend.

**Results:**

The overall AAMR exhibited a notable increase from 15.2 in 1999 to 24.9 in 2020. However, a recent decline was observed from 2013 to 2020 (APC: −2.1; 95% confidence interval [CI]: −3.4 to 0.65). African Americans experienced the highest mortality rate, surpassing that of Whites by more than twofold (AAMR: 20.3). Middle‐aged adults (35–54 years) faced the greatest mortality burden among all other age groups. Urban–rural disparities were significant, with urban areas showing substantially higher AAMRs compared to rural areas. Notably, urban AAMR decreased between 2013 and 2020 (APC: −2.7).

**Conclusion:**

The observed decrease in mortality related to cardiovascular disease and viral hepatitis over the past decade can be attributed to several factors, including heightened awareness and screening efforts, the introduction of novel and improved direct‐acting antiviral therapies, and the implementation of integrated public health models.

## 1 Introduction

According to the World Health Organization (WHO), viral hepatitis is the seventh leading cause of death worldwide and is the only communicable disease where mortality continues to rise, with up to 1.4 million deaths estimated globally [1, 2]. In the United States, 47 states reported approximately 13 300 infections of acute hepatitis B, while 40 states reported 14 229 incidents of chronic hepatitis B in 2021. Moreover, 43 states reported a staggering total of 107 540 newly diagnosed cases of chronic hepatitis C and an estimated 69 800 acute hepatitis C infections across 42 states [3]. Ultimately, these deaths can largely be attributed to viral hepatitis‐related cirrhosis and liver malignancy, making it an imminent public health concern.

A strong association exists between cardiovascular and liver disease, linked further by their common risk factors including dyslipidemia, diabetes mellitus, and atherosclerotic cardiovascular disease [4]. Regional differences in cardiovascular disease and liver cancer are also seen in Japan, with western Japan facing more issues comparatively [5]. Cirrhosis, a leading cause of mortality in hepatitis C patients, can lead to cirrhotic cardiomyopathy (CCM) which is found in up to 50% of patients suffering from cirrhosis [6, 7]. Numerous studies have suggested a positive correlation between carotid atherosclerosis and hepatitis C, as well as coronary artery disease and hepatitis B [8, 9, 10, 11, 12]. Additionally, cardiovascular disease stands as the primary cause of mortality worldwide, and has been the leading cause of death in the United States for the past century [13, 14].

Further adding to the urgency, while the “Viral Hepatitis National Progress Repor” was able to achieve or exceed its annual target in some categories, it failed to meet the overall 2025 outcome goals for new viral hepatitis infections and viral hepatitis‐related deaths, both overall and for key target populations [15]. On the background of these alarming findings, a thorough examination of current demographic and regional trends in cardiovascular and viral hepatitis‐related mortality is warranted. These insights will be invaluable for evidence‐based healthcare planning, resource allocation for viral hepatitis control, and preventive strategies in the United States.

## 2 Methodology

### 2.1 Study Setting and Population

We conducted a descriptive analysis using death certificate data sourced from the Centers for Disease Control and Prevention's WONDER (Wide‐Ranging Online Data for Epidemiologic Research) database [16]. Our primary objective was to assess the mortality rates among individuals diagnosed with cardiovascular disease with underlying viral hepatitis through a 21‐year period, ranging from 1999 to 2020. To identify these individuals, we employed the International Statistical Classification of Diseases and Related Health Problems, 11th Revision codes, B15, B16, B17, B18, and B19 for viral hepatitis, and I00‐I99 for cardiovascular disease, respectively [17, 18]. Our analysis focused on data retrieved from death certificates that were part of the Multiple Cause of Death Public Use dataset, which permitted the investigation of patient data that were identified to have cardiovascular disease with underlying viral hepatitis, either as a contributing factor or the primary cause of death in patients ≥ 15 years of age. We did not require institutional review board approval as we used a de‐identified government‐provided public‐use dataset following Strengthening the Reporting of Observational Studies in Epidemiology (STROBE) guidelines [19].

### 2.2 Data Abstraction

The data was categorized by abstracted demographic factors, including population size, age, gender, race/ethnicity, state, urbanization region, and place of death. Locations of death included inpatient, outpatient, emergency room, death on arrival, home, hospice/nursing home/long‐term care facility, or unknown. Racial/ethnic groups were defined as White, Black or African American, American Indian or Alaskan Native, and Asian or Pacific Islanders.

Age groups were stratified as 15–34, 35–54, and 55+ years. We categorized our study population geographically using the Urban–Rural Classification Scheme from the National Center for Health Statistics. Urban areas encompass metropolitan regions, ranging from small to medium metropolitan areas housing populations between 50 000 and 1 million to larger metropolitan cities with populations exceeding 1 million. Rural areas, in contrast, encompass locales with populations numbering less than 50 000. We also divided the United States into four regions based on the US Census Bureau's classification: Northeast, Midwest, South, and West [20].

### 2.3 Statistical Analysis

We analyzed the demographic and regional patterns of cardiovascular mortality with underlying viral hepatitis from 1999 to 2020, considering gender, race, age, urbanization, and census data. To quantify these patterns, we computed both crude and age‐adjusted mortality rates (AAMR) per 1 000 000 individuals, using the 2000 US population as a baseline for AAMR standardization. To assess the temporal changes in mortality rates, we used the Joinpoint Regression Program (Version 5.0.2, National Cancer Institute) [21]. This analytical approach involved fitting log‐linear regression models to crude data trends to calculate the annual percent change (APC) in AAMR with its 95% confidence interval (CI) using the Monte Carlo permutation test. To categorize APCs as increasing or decreasing, we relied on their statistical deviation from the null hypothesis of zero change. Statistical significance was determined using a two‐tailed *t*‐test with a threshold of *p* < 0.05.

## 3 Results

### 3.1 Overview of Patterns and Trends

A total of 138 416 cardiovascular‐related deaths in patients with viral hepatitis occurred between 1999 and 2020 among individuals (≥ 15 years old) (Table 1). Information on the location of death was available for 138 074 deaths. Of these known deaths, the vast majority (56.0%) occurred in medical facilities, followed by the decedent's home (26.4%), then nursing home/long‐term care (8.7%), then hospice facility (4.4%), and the fewest deaths occurred in other places (4.3%).

**TABLE 1:** Demographic characteristics of deaths due to in the United States from 1999 to 2020.

Variable	Deaths (%)	Deaths (*n*)	AAMR (PER 1000000)
Entire cohort	100%	138 416	23.2 (23.1–23.4)
Gender			
Female	30.0%	41 488	13.2 (13.1–13.3)
Male	70.0%	96 928	34.3 (34.1–34.5)
Census region			
Northeast	16.7%	23 052	20.6 (20.3–20.9)
Midwest	13.3%	18 378	14.0 (13.8–14.2)
South	37.2%	51 497	23.4 (23.2–23.6)
West	32.9%	45 489	34.5 (34.2–34.8)
Race/ethnicity			
American Indian or Alaska Native	1.5%	2022	35.7 (34.1–37.3)
Asian or Pacific Islander	4.6%	6298	25.5 (24.9–26.2)
Black or African American	21.7%	29 981	45.6 (45.1–46.2)
White	72.3%	100 115	20.3 (20.2–20.4)
Urbanization			
Urban	86.4%	119 580	24.0 (23.9–24.2)
Rural	13.6%	18 836	19.6 (19.3–19.9)
Age groups			
15–34 years	1.1%	1507	0.84 (0.80–0.89)
35–54 years	29.1%	40 316	20.4 (20.2–20.6)
55+ years	69.8%	96 593	55.9 (55.6–56.3)
Place of death^a^			
Medical facility	56.0%	77 550	—
Decedent's home	26.4%	36 502	—
Nursing home/long term care	8.7%	11 986	—
Hospice facility	4.4%	6116	—
Other	4.3%	5920	—
Place of death unknown	0.25%	342	—
Type of hepatitis			
Hepatitis A	0.86%	1213	0.20 (0.19–0.22)
Hepatitis B	10.3%	14 432	2.5 (2.5–2.5)
Hepatitis C	88.8%	124 784	20.9 (20.8–21.0)
Hepatitis D	—	—	—
Hepatitis E	0.021%	30	0.000 (0.000–0.000)

^a^ AAMR is not applicable for place of death.

**FIGURE 1:**
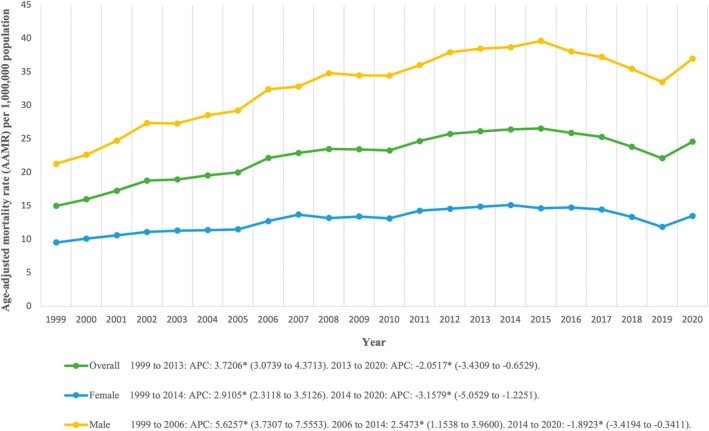
Trends in overall and sex‐stratified age‐adjusted cardiovascular mortality in patients with underlying viral hepatitis in the United States from 1999 to 2020. * Indicates the statistically significant difference of annual percentage change (APC) from 0 at α = 0.05.

The AAMR for cardiovascular‐related mortality in viral hepatitis in our study group (≥ 15 years old) was 23.2 from 1999 to 2020. The overall AAMR increased between 1999 and 2013 (APC: 3.7 [95% CI, 3.1 to 4.4]), followed by a decrease between 2013 and 2020 (APC: −2.1 [95% CI, −3.4 to 0.65]). Visual trends are illustrated in Figure 1 with comprehensive data available in Table S1 and Figure S1.

### 3.2 Demographics Trends

#### 3.2.1 Sex

During the study period of 1999 to 2020, the male AAMR was consistently higher than the female AAMR (overall AAMR for men: 34.3 [95% CI, 34.1–34.5]; for women: 13.2 [95% CI, 13.1–13.3]). An increasing trend was noted for men between 1999 and 2006 with an APC of 5.6 (95% CI, 3.7–7.6). It remained at 2.5 (95% CI, 1.2–4.0) between 2006 and 2014. A decrease in trends was noted between 2014 and 2020 with an APC of −1.8 (95% CI, −3.4–0.34). For women, an increasing trend was noted between 1999 and 2014 with an APC of 2.9 (95% CI, 2.3–3.5), followed by a decreasing trend between 2014 and 2020 with an APC of −3.2 (95% CI, −5.1 to −1.2). Visual trends are illustrated in Figure 1 with comprehensive data available in Table 2, Table S1, and Figure S1.

**TABLE 2:** Annual percentage changes (APCs) and average annual percentage changes (AAPCs) in mortality rate in the USA from 1999 to 2020.

Variable	Trend Segment	Lower endpoint	Upper endpoint	APC (95% CI)	AAPC (95% CI)	*p*
Entire cohort	1	1999	2013	3.7^a^ (3.1–4.4)	1.8^a^ (1.2–2.4)	< 0.000001
	2	2013	2020	−2.1^a^ (−3.4 to −0.65)		
Sex						
Female	1	1999	2014	2.9^a^ (2.3–3.5)	1.1^a^ (0.75–1.5)	< 0.000001
	2	2014	2020	−3.2^a^ (−5.1 to −1.2)		
Male	1	1999	2006	5.6^a^ (3.7–7.6)	2.3^a^ (1.4–3.1)	< 0.000001
	2	2006	2014	2.5^a^ (1.2–4.0)		
	3	2014	2020	−1.9^a^ (−3.4 to −0.34)		
US census region						
Northeast	1	1999	2012	1.9^a^ (0.78–2.9)	−0.96 (−1.9 to 0.0007)	0.050164
	2	2012	2020	−5.3^a^ (−7.3 to −3.3)		
Midwest	1	1999	2007	6.1^a^ (3.4–8.9)	3.2^a^ (2.1–4.3)	< 0.000001
	2	2007	2020	1.5^a^ (0.54–2.5)		
South	1	1999	2015	4.2^a^ (3.7–4.7)	2.9^a^ (2.4–3.5)	< 0.000001
	2	2015	2020	−1.1 (−3.0 to 0.83)		
West	1	1999	2012	4.2^a^ (3.5–5.0)	1.2^a^ (0.57–1.8)	0.000126
	2	2012	2020	−3.6^a^ (−4.8 to −2.5)		
Race/ethnicity						
American Indian or Alaska Native	1	2000	2020	4.0^a^ (3.0–5.1)	4.0^a^ (3.0–5.1)	< 0.000001
Asian or Pacific Islander	1	1999	2007	1.7 (−1.5 to 5.0)	−1.5^a^ (−2.8 to −0.21)	0.023666
	2	2007	2020	−3.4^a^ (−4.6 to −2.2)		
Black or African American	1	1999	2006	6.5^a^ (3.9–9.1)	1.9^a^ (0.77–3.1)	0.000957
	2	2006	2015	1.5 (−0.031 to 3.0)		
	3	2015	2020	−3.4^a^ (−6.0 to −0.66)		
White	1	1999	2013	3.9^a^ (3.4–4.5)	2.0^a^ (1.4–2.5)	< 0.000001
	2	2013	2020	−1.8^a^ (−3.1 to 0.58)		
Urbanization						
Urban	1	1999	2013	3.4^a^ (2.8–4.1)	1.4^a^ (0.75–2.0)	0.000010
	2	2013	2020	−2.7^a^ (−4.1 to −1.3)		
Rural	1	1999	2002	12.9^a^ (2.8–24.0)	4.9^a^ (3.5–6.4)	< 0.000001
	2	2002	2015	4.7^a^ (3.9–5.6)		
	3	2015	2020	0.93 (−1.6 to 3.5)		
Age						
15–34 years	1	1999	2011	−0.25 (−2.9 to 2.5)	2.9^a^ (0.83–4.9)	0.005504
	2	2011	2020	7.1^a^ (3.6–10.8)		
35–54 years	1	1999	2003	8.8^a^ (5.5–12.1)	−2.0^a^ (−3.1 to −0.95)	0.000243
	2	2003	2007	0.73 (−3.3 to 5.0)		
	3	2007	2015	−4.3^a^ (−5.5 to −3.2)		
	4	2015	2020	−8.5^a^ (−10.7 to −6.1)		
55+ years	1	1999	2014	6.3^a^ (5.6–6.9)	4.2^a^ (3.6–4.8)	< 0.000001
	2	2014	2020	−0.85 (−2.5 to 0.79)		
Hepatitis type						
Hepatitis A	1	1999	2007	−14.0^a^ (−17.0 to 10.8)	−3.9^a^ (−6.8 to −0.83)	0.012923
	2	2007	2015	−5.3 (−10.9 to 0.56)		
	3	2015	2020	17.8^a^ (7.9–28.5)		
Hepatitis B	1	1999	2018	−1.5^a^ (−1.9 to −1.2)	−0.56 (−1.6 to 0.49)	0.294590
	2	2018	2020	9.2 (−2.4 to 22.2)		
Hepatitis C	1	1999	2007	6.1^a^ (4.5–7.8)	2.2^a^ (1.2–3.1)	0.000003
	2	2007	2015	2.0^a^ (0.57–3.5)		
	3	2015	2020	−3.7^a^ (−5.9 to −1.5)		

^a^ Indicates that the AAPC is significantly different from zero at the alpha = 0.05 level.

#### 3.2.2 Age

An increasing mortality trend was noted with increasing age. For the 15–34 years age group, the AAMR was 0.84 (95% CI, 0.80–0.89). For the 35–54 years age group, the AAMR was 20.4 (95% CI, 20.2–20.6). For the 55+ years age group, the AAMR was 55.9 (95% CI, 55.6–56.3). A prominent disparity was observed among different age groups. In the 15–34 years group, there was a decreasing trend from 1999 to 2011 with an APC of −0.25 (95% CI, −2.9 to 2.5), followed by an increasing trend up to 2020 with an APC of 7.1 (95% CI, 3.6–10.8).

In the 35–54 years group, there was an increasing trend between 1999 and 2003, with an APC of 8.8 (95% CI 5.5–12.1). The APC was 0.73 (95% CI −3.3 to 5.0) between 2003 and 2007, followed by a decreasing mortality trend with an APC of −4.3 (95% CI −5.5 to −3.2) between 2007 and 2015 and −8.5 (95% CI −10.7 to −6.1) up to 2020.

In the 55+ years group, there was an increasing trend between 1999 and 2014 with an APC of 6.3 (95% CI 5.6–6.9), followed by a decreasing trend up to 2020 with an APC of −0.85 (95% CI −2.5 to 0.79). Comprehensive data is available in Table 2.

#### 3.2.3 Race and Ethnicity

NH Black or African American had the highest overall AAMR among all ethnicities (AAMR: 45.6 [95% CI, 45.1–46.2]). They were followed by American Indian or Alaska Native (AAMR: 35.7 [95% CI, 34.1–37.3]), Asian or Pacific Islander (AAMR: 25.5 [95% CI, 24.9–26.2]), and White (AAMR: 20.3 [95% CI, 20.2–20.4]).

The increasing and decreasing trends of AAMR were quite different for different ethnicities. Among the Black or African American population, there was an increasing AAMR trend from 1999 to 2006, with an APC of 6.5 (95% CI, 3.9–9.1). From 2006 to 2015, the APC was 1.5 (95% CI, −0.031 to 3.0), followed by a decreasing trend in AAMR from 2015 to 2020, with an APC of −3.4 (95% CI, −6.0 to −0.66).

**FIGURE 2:**
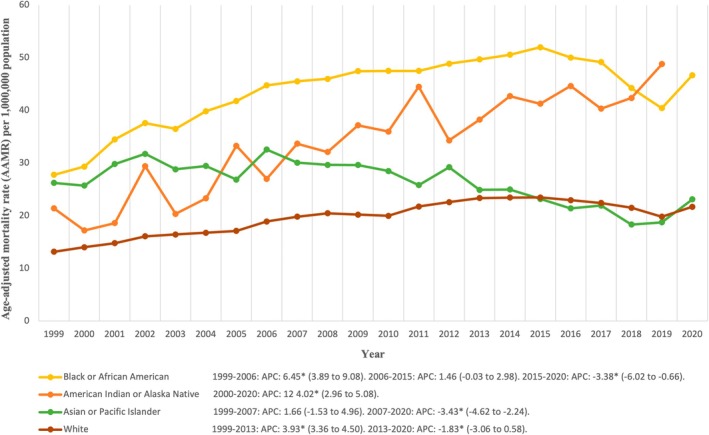
Trends in cardiovascular mortality in patients with underlying viral hepatitis stratified by ethnicity in the United States from 1999 to 2020. * Indicates the statistically significant difference of annual percentage change (APC) from 0 at α = 0.05.

Among American Indian or Alaska Natives, only an increasing mortality trend was noted from 2000 to 2020 with an APC of 4.0 (95% CI, 3.0–5.1). Among Asian or Pacific Islanders, an increasing mortality trend was noted between 1999 and 2007 with an APC of 1.7 (95% CI, 1.5–5.0), followed by a decreasing trend between 2007 and 2020 with an APC of −3.4 (95% CI, −4.6 to −2.2). Among White individuals, an increasing mortality trend was noted between 1999 and 2013 with an APC of 3.9 (95% CI, 3.4–4.5), followed by a decreasing trend between 2013 and 2020 with an APC of −1.8 (95% CI, −3.1 to 0.58). Visual trends are illustrated in Figure 2 with comprehensive data available in Tables 2, S2, and Figure S2.

### 3.3 Geographical Patterns and Regional Variation

#### 3.3.1 Urban–Rural

The mortality rate was higher in urban areas compared to rural areas. The AAMR for urban areas was 24.0 (95% CI, 23.9–24.2), while the AAMR for rural areas was 19.6 (95% CI, 19.3–19.9). The mortality trends were also quite different for urban and rural areas.

In urban areas, there was an increasing trend between 1999 and 2013 with an APC of 3.44 (95% CI, 2.8 to 4.1), followed by a decreasing trend from 2013 to 2020 with an APC of −2.7 (95% CI, −4.1 to −1.3). In rural areas, there was an increasing trend; from 1999 to 2002, the APC was 12.9 (95% CI, 2.8 to 24.0), from 2002 to 2015, the APC was 4.7 (95% CI, 3.9 to 5.6), and from 2015 to 2020, the APC was 0.93 (95% CI, −1.6 to 3.5). Further details regarding the urban–rural trends are available in Tables 2, S4, and Figure S3.

#### 3.3.2 Census Regions

A prominent disparity in AAMR was observed among different census regions. The West had the highest AAMR of 34.5 (95% CI, 34.2–34.8), followed by the South with 23.4 (95% CI, 23.2–23.6), Northeast with 20.6 (95% CI, 20.3–20.9), and Midwest with 14.0 (95% CI, 13.8–14.2).

An increasing mortality trend was noted in the West between 1999 and 2012 with an APC of 4.2 (95% CI, 3.5–5.0), followed by a decreasing trend up to 2020 with an APC of −3.6 (95% CI, −4.8 to 2.5). Similar trends were noted in the South with an increasing trend from 1999 to 2015 with an APC of 4.2 (95% CI, 3.7–4.7), followed by a decreasing trend up to 2020 with an APC of −1.1 (95% CI, −3.0 to 0.8). Similar trends were noted in the Northeast as well, with an increasing trend between 1999 and 2012 with an APC of 1.8 (95% CI, 0.78–2.9), followed by a decreasing trend up to 2020 with an APC of −5.3 (95% CI, −7.3 to 3.3). In the Midwest, an increasing trend was noted with an APC of 6.1 (95% CI, 3.4–8.9) between 1999 and 2007, followed by an APC of 1.5 (95% CI, 0.54–2.5) between 2007 and 2020.

**FIGURE 3:**
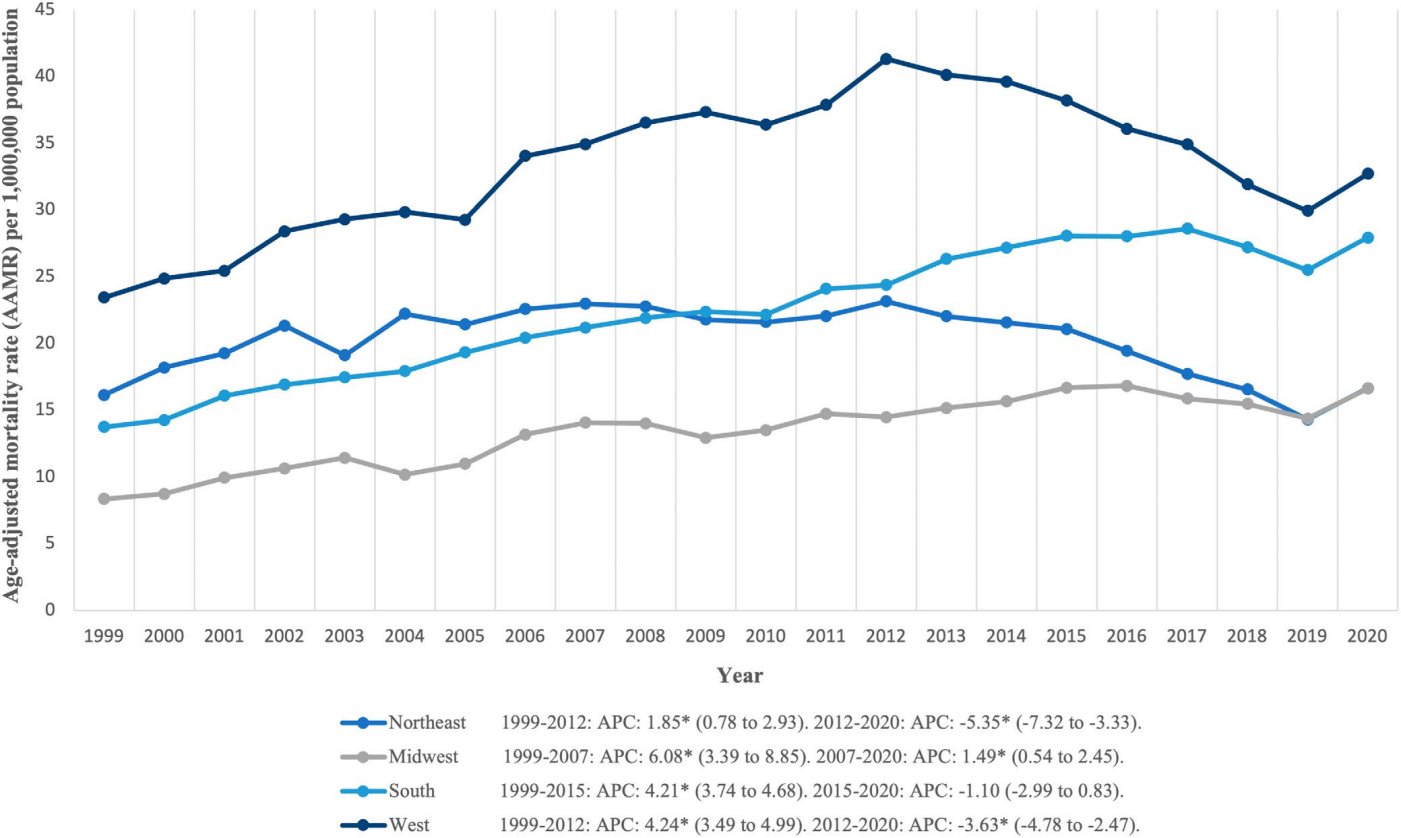
Trends in cardiovascular mortality in patients with underlying viral hepatitis stratified by census regions in the United States from 1999 to 2020. * Indicates the statistically significant difference of annual percentage change (APC) from 0 at α = 0.05.

Visual trends are illustrated in Figure 3, and comprehensive analysis data is available in Tables 2, S3, and Figure S4.

### 3.4 Trends in Different Viral Hepatitis Serotypes

Our analysis found that mortality trends for different serotypes of viral hepatitis vary significantly. A decreasing trend was noted for hepatitis A between 1999 and 2007, with an APC of −14.0 (95% CI, −17.0 to 10.9). It remained at −5.3 (95% CI, −10.9 to 0.56) between 2007 and 2015. An increasing trend was noted between 2015 and 2020, with an APC of 17.8 (95% CI, 7.9–28.5). For hepatitis B, there was a decrease in mortality trends between 1999 and 2018, with an APC of −1.5 (95% CI, −1.9 to −1.2), followed by an increasing trend between 2018 and 2020, with an APC of 9.2 (95% CI, −2.4 to 22.2). An increasing trend was noted for hepatitis C between 1999 and 2007, with an APC of 6.1 (95% CI, 4.5–7.8). It remained at 2.0 (95% CI, 0.57–3.5) between 2007 and 2015. A decreasing trend was noted between 2015 and 2020, with an APC of −3.7 (95% CI, −5.9 to −1.5). Summarized details comparing the cardiovascular mortality patterns of different serotypes are available in Tables 2, S5, and Figure S5.

## 4 Discussion

Using national death certificate data, we have identified several distinct patterns in mortality related to viral hepatitis over the past 20 years. While overall mortality due to cardiovascular‐related issues in viral hepatitis has declined, there are notable variations across different serotypes. Specifically, viral hepatitis A and B have experienced a surge in mortality over the past 5 years. Demographic disparities have also emerged. Males and Black or African American adults appear to be disproportionately affected. Additionally, urban–rural differences exist, with a higher burden of mortality observed in urban areas compared to rural ones. This disparity has widened over time. Furthermore, mortality rates vary significantly across states and geographical regions, highlighting the need for targeted interventions and public health strategies.

Despite the overall population‐level benefits, patients with viral hepatitis continue to experience excess all‐cause and cardiovascular mortality. Even in the context of hepatitis C, where an overall decrease in cardiovascular‐related mortality burden has been observed, data from other studies suggest that cardiovascular events still pose a significant risk for patients with hepatitis C [1]. Our findings reveal an overall increase in cardiovascular‐related mortality associated with viral hepatitis from 1999 to 2013. Several factors may contribute to this early increase in mortality, including a rise in the prevalence of viral hepatitis, improved case detection and reporting, as well as limited adoption of antiviral therapies for viral hepatitis. The subsequent decline (2013–2020) can be attributed to advancements in healthcare, including easier access to antiviral therapies and the development of new direct‐acting antivirals (DAAs). Additionally, urban populations may have benefited earlier from public health campaigns, such as Medicaid expansions, improving treatment access post‐2013. Early detection and effective management of cardiovascular complications have also played a role in reducing mortality during this period [7, 8, 22].

The further decline could be attributed to the “International Viral Hepatitis Elimination Movement”, launched by the WHO that seeks to irradiate hepatitis B and C associated infections by 2030 [23]. In the United States, the “National Viral Hepatitis Action Plan,” initiated in 2011, has been instrumental in screening, diagnosing, and treating hepatitis C. The Department of Veterans Affairs has taken a lead role in these efforts, starting from 2014, and this concerted approach may have contributed to the reduction in hepatitis C‐associated mortality [24].

The most recent drop in hepatitis B cases could also be due to the COVID‐19 pandemic, which on a positive note resulted in reduced gatherings and simultaneous risk of drug use, but on a bleaker note resulted in a reduction in possible diagnosis and surveillance of viral hepatitis cases due to disruptions in healthcare services and facilities and the strained economy combined with the increased deployment, which led to patient delay in seeking medical attention [25].

Further, the population attributable risk of cardiovascular mortality in patients seropositive for hepatitis C was higher compared to those who were seronegative [26], as well as in those who were co‐infected with HIV and hepatitis C [27, 28], further highlighting the importance of early and aggressive viral hepatitis management as means to decrease population‐level cardiovascular mortality.

An updated estimate of the cardiovascular disease and viral hepatitis mortality burden is crucial for informing policy measures and identifying focus areas for targeted interventions. In our current study, we report that while there has been an overall decrease in cardiovascular‐related mortality rates associated with hepatitis A and hepatitis B over the last two decades, a recent increase starting in 2014 threatens years of progress. Notably, this trend is more pronounced among Black and African American males compared to females and individuals of Asian and Pacific Islander and White populations.

Even after HBV control or HCV eradication, steatotic liver disease (MASLD/MAFLD) can develop and remains a significant risk factor for both cardiovascular mortality and HCC. Studies have shown that MASLD is associated with HCC development post‐SVR in HCV patients, even with low AFP levels. MAFLD also contributes to hepatocarcinogenesis in HBV patients with undetectable viral loads and acts as a co‐factor in mixed‐etiology HCC. These findings highlight the need to monitor metabolic dysfunction even after viral suppression [29, 30, 31, 32].

MASLD is increasingly recognized as a key contributor to cardiovascular disease, and future strategies must address its systemic impact beyond the liver. Elevated ALT levels (> 30 U/L) and MASLD have been linked to significant hepatic fibrosis in older adults (≥ 65 years), underscoring the need for early detection and intervention in this population. Moreover, MASLD—particularly when co‐existing with alcohol‐related liver disease (ALD)—has been independently associated with chronic kidney disease (CKD), highlighting its multisystem involvement. Recent findings also suggest that prolonged antibiotic exposure may increase cardiovascular risk in MASLD patients, pointing to the complex interplay between the gut microbiome, metabolic dysfunction, and cardiovascular outcomes. These insights emphasize the need for comprehensive management strategies that target both hepatic and extrahepatic manifestations of MASLD [33, 34, 35, 36].

The observed gender difference can be attributed to various risk factors. Estrogen, which has protective effects in women, may play a role in mitigating cardiovascular risk [37, 38], healthcare utilization, and treatment outcomes, emphasizing the importance of a tailored approach to cardiovascular disease prevention and management based on gender. In contrast, males exhibit behavioral predispositions that contribute to the development of both hepatitis B and hepatitis C as well as heart disease. These risk factors include smoking, alcohol use, and sharing infected needles among intravenous drug users, particularly for hepatitis C. Given their higher prevalence in men, these behaviors increase the risk of both viral hepatitis and cardiovascular events [39, 40, 41]. Furthermore, the prevalence of HIV is higher among Black or African American males compared to males in general. This association may contribute to the increased cardiovascular mortality observed in individuals suffering from both HIV and viral hepatitis [35, 36, 42, 43].

Our study reveals significant ethnic and regional variations in cardiovascular‐associated mortality rates among individuals with viral hepatitis. Specifically, Black or African American individuals exhibit the highest overall AAMR, followed by American Indian or Alaska Natives, Asian or Pacific Islanders, and White individuals. These findings align with insights from the REACH registry [34], which also reported a greater cardiovascular disease burden in Black populations compared to other ethnic groups.

The observed disparities likely stem from underlying social determinants of health. Factors such as access to healthcare, socioeconomic status, and environmental conditions contribute to differential risk and outcomes for cardiovascular disease across diverse populations [44]. However, recent reports from the CDC suggest that the disparities between White and Black individuals have narrowed in recent years [45].

Starting in 2011, we observed a sharp increase in mortality rates related to cardiovascular disease and viral hepatitis in the younger age group (15–34 years old), contrasting with a decrease in mortality among middle‐aged to older individuals (55–69 years old) over the past 5 years. Several factors may contribute to this alarming trend, including rising rates of obesity, smoking, substance use, and other cardiovascular comorbidities among young adults [46]. Additionally, the well‐established epidemic of drug use among the younger population (15–50 years old) is closely linked to hepatitis B and C infections, where hematogenous transmission is the most common route of infection [47, 48].

Despite ongoing efforts by the WHO to achieve its “2030 Global Viral Hepatitis eradication” plan, significant challenges persist. Unfortunately, the number of hepatitis B‐related morbidity and mortality cases continues to rise in Africa and Asia, remaining a consistent cause of annual deaths in the United Kingdom and the United States [34].

Our own findings highlight prominent disparities in AAMR between urban and rural populations. Urban areas exhibit higher AAMR compared to rural areas, reflecting complex factors such as chronic disease prevalence, poverty, fragmented healthcare, and limited access to preventative and specialist care in rural regions [46]. Furthermore, geographic differences are evident across different census regions. The Western regions of the US have the highest AAMR, followed by the South, Northeast, and Midwest.

These variations underscore the need for targeted interventions to address the increased cardiovascular mortality burden in the Western states [49].

State‐level differences in cardiovascular disease and viral hepatitis‐related mortality rates can be attributed to several factors. These include variations in the demographic composition of states, inherent disease risks, and heterogeneities in metabolic, behavioral, and clinical risk factors faced by residents. Additionally, state‐specific legislation, such as healthcare expenditure and Medicaid expansion, directly impacts mortality rates in these regions. Viral hepatitis A and E primarily spread through fecal‐oral routes. Improving hygienic and sanitary conditions can play a crucial role in preventing their transmission and subsequent mortality [50]. Urban areas show a higher prevalence of viral hepatitis due to denser populations, increased IV drug abuse, and transmission risks (e.g., homelessness), while also potentially benefiting from earlier diagnosis and reporting through their healthcare facilities, ultimately leading to higher recorded mortality rates [51]. Moreover, urban areas face greater cardiovascular risks due to increased rates of comorbidities, including hypertension and diabetes mellitus, resulting in a rise in cardiovascular mortality [52]. In contrast, rural healthcare typically faces disparities in access to specialists, leading to underdiagnosis and delayed treatment.

While newer combinations of direct‐acting antivirals, such as NS3/4A protease inhibitors like sofosbuvir and NS5 replication complex inhibitors, have demonstrated promising results in reducing viral hepatitis‐related mortality, their high cost remains a barrier for a significant portion of society [49]. Nucleos(t)ide analogs reduce HBV‐related mortality by suppressing viral replication and preventing cirrhosis, indirectly lowering cardiovascular risks. However, their impact on AAMRs is nuanced because the CDC‐WONDER dataset lacks granularity on antiviral use, although cohort studies suggest nucleos(t)ide analogs reduce liver‐related mortality [53]. Regional disparities in DAA access reveal distinct patterns, including Western/Southern states showing higher AAMRs, correlating with later DAA adoption due to restrictive Medicaid policies. Meanwhile, the Midwestern regions demonstrate earlier DAA uptake, contributing to steeper declines in HCV‐related mortality post‐2014. This is in addition to the barriers faced by rural populations that disproportionately affect these regions, exacerbating the urban–rural divide [54]. Federal and state‐level legislative decisions play a pivotal role in ensuring equitable access to life‐saving therapies and mitigating the increasing burden of mortality.

### 4.1 Clinical Implications and Future Recommendations

Overall, our study provides valuable insights into the epidemiology of cardiovascular mortality among individuals with viral hepatitis, highlighting the need for targeted strategies to address the evolving burden of cardiovascular disease in this population. Targeted interventions should be arranged for specific genders, ethnicities, and regions. Future research should focus on explaining the underlying mechanisms responsible for the observed trends and disparities, as well as evaluating the effectiveness of interventions aimed at reducing cardiovascular morbidity and mortality among individuals with viral hepatitis. Developing elaborate frameworks such as the Lean management strategy, “Hepatitis Innovation Teams” (HIT) collaborative leadership could prove to be the game‐changer in eradicating hepatitis B and C in the Eastern and African countries [24, 46]. By addressing the challenges of cardiovascular‐related mortality in patients with viral hepatitis, we can work toward achieving equitable health outcomes for all populations affected by these conditions.

### 4.2 Limitations

This study has several limitations. First, since the information available on the CDC‐WONDER database is derived from death certificates and classified using ICD codes, any missed or inaccurate diagnoses can result in the incorrect categorization of cardiovascular disease or viral hepatitis as causes of mortality. Second, categories of race/ethnicity can be identified incorrectly, leading to misrepresentation of data [55]. Third, variations in the accuracy and completeness of data may introduce reporting and coding biases. Last, information on socioeconomic determinants of health was unavailable, which might impact access to care. Additionally, greater research into prevalence, social determinants, and any potential treatment‐related disparities that have not been explored in our analysis needed.

## 5 Conclusions

This wide‐ranging comprehensive analysis of contemporary US‐based data trends of cardiovascular mortality in patients with underlying viral hepatitis revealed several important findings. Despite the decrease in mortality patterns throughout the recent years, the male sex and the Black or African American population appear to be disproportionately affected. Additionally, urban–rural disparities continue to persist, with a greater burden observed in urbanized regions. Furthermore, a surge in hepatitis A and B‐related mortality has been identified. In conclusion, although commendable screening and treatment efforts have been visualized, targeted therapy is paramount in addressing the persistent demographic and regional disparities. Greater emphasis on public health preventative strategies may be essential in curbing the recent rise in hepatitis A and B‐related mortality trends. However, further research is demanded in order to delve deeper into the socio‐economic differences (e.g., education status, income level, neighborhood) as well as treatment‐related outcome analysis.

## Ethics Statement

The authors have nothing to report.

## Conflicts of Interest

The authors declare no conflicts of interest.

## Supporting information


**Data S1.** Supporting Information.

## Data Availability

The data that supports the findings of this study are available in the Supporting Information of this article.
