# Supplementary material for: Temporal Trends in Cardiovascular Mortality in Underlying Viral Hepatitis: A Retrospective Analysis of Gender, Racial/Ethnic, and Regional Disparities
Source: JGH Open. 2025 Jul 27;9(8):e70235. doi: 10.1002/jgh3.70235 (PMC12301567; doi:10.1002/jgh3.70235)
Supplement: Supplementary file 1 — Data S1. Supporting Information. [file JGH3-9-e70235-s001.docx]

| **Gender** | **Year** | **Age Adjusted Rate** |
| --- | --- | --- |
| Female | 1999 | 9.552 |
| Female | 2000 | 10.115 |
| Female | 2001 | 10.615 |
| Female | 2002 | 11.112 |
| Female | 2003 | 11.314 |
| Female | 2004 | 11.389 |
| Female | 2005 | 11.499 |
| Female | 2006 | 12.749 |
| Female | 2007 | 13.719 |
| Female | 2008 | 13.209 |
| Female | 2009 | 13.417 |
| Female | 2010 | 13.147 |
| Female | 2011 | 14.286 |
| Female | 2012 | 14.583 |
| Female | 2013 | 14.89 |
| Female | 2014 | 15.131 |
| Female | 2015 | 14.655 |
| Female | 2016 | 14.772 |
| Female | 2017 | 14.471 |
| Female | 2018 | 13.376 |
| Female | 2019 | 11.88 |
| Female | 2020 | 13.52 |
| Male | 1999 | 21.301 |
| Male | 2000 | 22.647 |
| Male | 2001 | 24.746 |
| Male | 2002 | 27.392 |
| Male | 2003 | 27.311 |
| Male | 2004 | 28.554 |
| Male | 2005 | 29.253 |
| Male | 2006 | 32.428 |
| Male | 2007 | 32.835 |
| Male | 2008 | 34.827 |
| Male | 2009 | 34.495 |
| Male | 2010 | 34.476 |
| Male | 2011 | 36.041 |
| Male | 2012 | 37.951 |
| Male | 2013 | 38.482 |
| Male | 2014 | 38.706 |
| Male | 2015 | 39.651 |
| Male | 2016 | 38.07 |
| Male | 2017 | 37.244 |
| Male | 2018 | 35.475 |
| Male | 2019 | 33.499 |
| Male | 2020 | 36.999 |
| Overall | 1999 | 15.009 |
| Overall | 2000 | 16.007 |
| Overall | 2001 | 17.282 |
| Overall | 2002 | 18.786 |
| Overall | 2003 | 18.962 |
| Overall | 2004 | 19.56 |
| Overall | 2005 | 19.998 |
| Overall | 2006 | 22.164 |
| Overall | 2007 | 22.931 |
| Overall | 2008 | 23.505 |
| Overall | 2009 | 23.457 |
| Overall | 2010 | 23.289 |
| Overall | 2011 | 24.687 |
| Overall | 2012 | 25.763 |
| Overall | 2013 | 26.152 |
| Overall | 2014 | 26.418 |
| Overall | 2015 | 26.582 |
| Overall | 2016 | 25.903 |
| Overall | 2017 | 25.31 |
| Overall | 2018 | 23.849 |
| Overall | 2019 | 22.123 |
| Overall | 2020 | 24.606 |

**Supplementary Table 1:** Overall and sex-stratified age-adjusted mortality rate (AAMR) for cardiovascular mortality and viral hepatitis in US adults from 1999 to 2020.

| **Race** | **Year** | **Age Adjusted Rate** |
| --- | --- | --- |
| American Indian or Alaska Native | 2000 | 21.4 |
| American Indian or Alaska Native | 2001 | 17.218 |
| American Indian or Alaska Native | 2002 | 18.608 |
| American Indian or Alaska Native | 2003 | 29.407 |
| American Indian or Alaska Native | 2004 | 20.368 |
| American Indian or Alaska Native | 2005 | 23.307 |
| American Indian or Alaska Native | 2006 | 33.302 |
| American Indian or Alaska Native | 2007 | 27.006 |
| American Indian or Alaska Native | 2008 | 33.684 |
| American Indian or Alaska Native | 2009 | 32.112 |
| American Indian or Alaska Native | 2010 | 37.17 |
| American Indian or Alaska Native | 2011 | 36.003 |
| American Indian or Alaska Native | 2012 | 44.512 |
| American Indian or Alaska Native | 2013 | 34.309 |
| American Indian or Alaska Native | 2014 | 38.244 |
| American Indian or Alaska Native | 2015 | 42.715 |
| American Indian or Alaska Native | 2016 | 41.278 |
| American Indian or Alaska Native | 2017 | 44.653 |
| American Indian or Alaska Native | 2018 | 40.349 |
| American Indian or Alaska Native | 2019 | 42.361 |
| American Indian or Alaska Native | 2020 | 48.807 |
| Asian or Pacific Islander | 1999 | 26.248 |
| Asian or Pacific Islander | 2000 | 25.724 |
| Asian or Pacific Islander | 2001 | 29.828 |
| Asian or Pacific Islander | 2002 | 31.754 |
| Asian or Pacific Islander | 2003 | 28.839 |
| Asian or Pacific Islander | 2004 | 29.461 |
| Asian or Pacific Islander | 2005 | 26.869 |
| Asian or Pacific Islander | 2006 | 32.567 |
| Asian or Pacific Islander | 2007 | 30.073 |
| Asian or Pacific Islander | 2008 | 29.671 |
| Asian or Pacific Islander | 2009 | 29.644 |
| Asian or Pacific Islander | 2010 | 28.515 |
| Asian or Pacific Islander | 2011 | 25.829 |
| Asian or Pacific Islander | 2012 | 29.221 |
| Asian or Pacific Islander | 2013 | 24.911 |
| Asian or Pacific Islander | 2014 | 24.989 |
| Asian or Pacific Islander | 2015 | 23.193 |
| Asian or Pacific Islander | 2016 | 21.398 |
| Asian or Pacific Islander | 2017 | 21.936 |
| Asian or Pacific Islander | 2018 | 18.328 |
| Asian or Pacific Islander | 2019 | 18.749 |
| Asian or Pacific Islander | 2020 | 23.128 |
| Black or African American | 1999 | 27.788 |
| Black or African American | 2000 | 29.326 |
| Black or African American | 2001 | 34.492 |
| Black or African American | 2002 | 37.591 |
| Black or African American | 2003 | 36.506 |
| Black or African American | 2004 | 39.854 |
| Black or African American | 2005 | 41.778 |
| Black or African American | 2006 | 44.771 |
| Black or African American | 2007 | 45.545 |
| Black or African American | 2008 | 45.993 |
| Black or African American | 2009 | 47.464 |
| Black or African American | 2010 | 47.516 |
| Black or African American | 2011 | 47.504 |
| Black or African American | 2012 | 48.894 |
| Black or African American | 2013 | 49.701 |
| Black or African American | 2014 | 50.577 |
| Black or African American | 2015 | 52.008 |
| Black or African American | 2016 | 50.032 |
| Black or African American | 2017 | 49.186 |
| Black or African American | 2018 | 44.251 |
| Black or African American | 2019 | 40.432 |
| Black or African American | 2020 | 46.683 |
| White | 1999 | 13.164 |
| White | 2000 | 14.02 |
| White | 2001 | 14.803 |
| White | 2002 | 16.099 |
| White | 2003 | 16.445 |
| White | 2004 | 16.78 |
| White | 2005 | 17.123 |
| White | 2006 | 18.915 |
| White | 2007 | 19.825 |
| White | 2008 | 20.47 |
| White | 2009 | 20.232 |
| White | 2010 | 19.984 |
| White | 2011 | 21.753 |
| White | 2012 | 22.596 |
| White | 2013 | 23.368 |
| White | 2014 | 23.445 |
| White | 2015 | 23.479 |
| White | 2016 | 22.957 |
| White | 2017 | 22.433 |
| White | 2018 | 21.526 |
| White | 2019 | 19.824 |
| White | 2020 | 21.705 |

**Supplementary Table 2:** Race-stratified age-adjusted mortality rate (AAMR) for cardiovascular mortality and viral hepatitis in US adults from 1999 to 2020.

| **Census Region** | **Year** | **Age Adjusted Rate** |
| --- | --- | --- |
| Census Region 1: Northeast | 1999 | 16.141 |
| Census Region 1: Northeast | 2000 | 18.202 |
| Census Region 1: Northeast | 2001 | 19.26 |
| Census Region 1: Northeast | 2002 | 21.342 |
| Census Region 1: Northeast | 2003 | 19.122 |
| Census Region 1: Northeast | 2004 | 22.225 |
| Census Region 1: Northeast | 2005 | 21.429 |
| Census Region 1: Northeast | 2006 | 22.587 |
| Census Region 1: Northeast | 2007 | 22.981 |
| Census Region 1: Northeast | 2008 | 22.78 |
| Census Region 1: Northeast | 2009 | 21.793 |
| Census Region 1: Northeast | 2010 | 21.615 |
| Census Region 1: Northeast | 2011 | 22.055 |
| Census Region 1: Northeast | 2012 | 23.157 |
| Census Region 1: Northeast | 2013 | 22.042 |
| Census Region 1: Northeast | 2014 | 21.582 |
| Census Region 1: Northeast | 2015 | 21.085 |
| Census Region 1: Northeast | 2016 | 19.45 |
| Census Region 1: Northeast | 2017 | 17.724 |
| Census Region 1: Northeast | 2018 | 16.564 |
| Census Region 1: Northeast | 2019 | 14.3 |
| Census Region 1: Northeast | 2020 | 16.651 |
| Census Region 2: Midwest | 1999 | 8.357 |
| Census Region 2: Midwest | 2000 | 8.751 |
| Census Region 2: Midwest | 2001 | 9.959 |
| Census Region 2: Midwest | 2002 | 10.657 |
| Census Region 2: Midwest | 2003 | 11.456 |
| Census Region 2: Midwest | 2004 | 10.185 |
| Census Region 2: Midwest | 2005 | 10.999 |
| Census Region 2: Midwest | 2006 | 13.175 |
| Census Region 2: Midwest | 2007 | 14.06 |
| Census Region 2: Midwest | 2008 | 14.02 |
| Census Region 2: Midwest | 2009 | 12.938 |
| Census Region 2: Midwest | 2010 | 13.5 |
| Census Region 2: Midwest | 2011 | 14.743 |
| Census Region 2: Midwest | 2012 | 14.475 |
| Census Region 2: Midwest | 2013 | 15.17 |
| Census Region 2: Midwest | 2014 | 15.661 |
| Census Region 2: Midwest | 2015 | 16.674 |
| Census Region 2: Midwest | 2016 | 16.831 |
| Census Region 2: Midwest | 2017 | 15.872 |
| Census Region 2: Midwest | 2018 | 15.469 |
| Census Region 2: Midwest | 2019 | 14.372 |
| Census Region 2: Midwest | 2020 | 16.63 |
| Census Region 3: South | 1999 | 13.744 |
| Census Region 3: South | 2000 | 14.26 |
| Census Region 3: South | 2001 | 16.082 |
| Census Region 3: South | 2002 | 16.92 |
| Census Region 3: South | 2003 | 17.463 |
| Census Region 3: South | 2004 | 17.936 |
| Census Region 3: South | 2005 | 19.331 |
| Census Region 3: South | 2006 | 20.445 |
| Census Region 3: South | 2007 | 21.192 |
| Census Region 3: South | 2008 | 21.908 |
| Census Region 3: South | 2009 | 22.388 |
| Census Region 3: South | 2010 | 22.162 |
| Census Region 3: South | 2011 | 24.104 |
| Census Region 3: South | 2012 | 24.391 |
| Census Region 3: South | 2013 | 26.346 |
| Census Region 3: South | 2014 | 27.186 |
| Census Region 3: South | 2015 | 28.074 |
| Census Region 3: South | 2016 | 28.034 |
| Census Region 3: South | 2017 | 28.614 |
| Census Region 3: South | 2018 | 27.232 |
| Census Region 3: South | 2019 | 25.511 |
| Census Region 3: South | 2020 | 27.949 |
| Census Region 4: West | 1999 | 23.453 |
| Census Region 4: West | 2000 | 24.873 |
| Census Region 4: West | 2001 | 25.455 |
| Census Region 4: West | 2002 | 28.423 |
| Census Region 4: West | 2003 | 29.32 |
| Census Region 4: West | 2004 | 29.866 |
| Census Region 4: West | 2005 | 29.283 |
| Census Region 4: West | 2006 | 34.059 |
| Census Region 4: West | 2007 | 34.949 |
| Census Region 4: West | 2008 | 36.555 |
| Census Region 4: West | 2009 | 37.35 |
| Census Region 4: West | 2010 | 36.414 |
| Census Region 4: West | 2011 | 37.895 |
| Census Region 4: West | 2012 | 41.345 |
| Census Region 4: West | 2013 | 40.147 |
| Census Region 4: West | 2014 | 39.634 |
| Census Region 4: West | 2015 | 38.22 |
| Census Region 4: West | 2016 | 36.095 |
| Census Region 4: West | 2017 | 34.932 |
| Census Region 4: West | 2018 | 31.936 |
| Census Region 4: West | 2019 | 29.951 |
| Census Region 4: West | 2020 | 32.733 |

**Supplementary Table 3**. Age-adjusted mortality rate (AAMR) stratified by census region for cardiovascular mortality and viral hepatitis in US adults from 1999 to 2020.

| **Urbanization** | **Year** | **Age Adjusted Rate** |
| --- | --- | --- |
| Urban | 1999 | 16.128 |
| Urban | 2000 | 17.396 |
| Urban | 2001 | 18.29 |
| Urban | 2002 | 19.912 |
| Urban | 2003 | 20.027 |
| Urban | 2004 | 20.698 |
| Urban | 2005 | 21.053 |
| Urban | 2006 | 23.273 |
| Urban | 2007 | 24.225 |
| Urban | 2008 | 24.502 |
| Urban | 2009 | 24.417 |
| Urban | 2010 | 24.255 |
| Urban | 2011 | 25.743 |
| Urban | 2012 | 26.71 |
| Urban | 2013 | 26.865 |
| Urban | 2014 | 27.328 |
| Urban | 2015 | 26.906 |
| Urban | 2016 | 26.294 |
| Urban | 2017 | 25.417 |
| Urban | 2018 | 23.825 |
| Urban | 2019 | 21.768 |
| Urban | 2020 | 24.442 |
| Rural | 1999 | 9.842 |
| Rural | 2000 | 9.501 |
| Rural | 2001 | 12.546 |
| Rural | 2002 | 13.324 |
| Rural | 2003 | 13.968 |
| Rural | 2004 | 14.214 |
| Rural | 2005 | 15.238 |
| Rural | 2006 | 17.072 |
| Rural | 2007 | 16.748 |
| Rural | 2008 | 18.958 |
| Rural | 2009 | 19.065 |
| Rural | 2010 | 18.656 |
| Rural | 2011 | 19.791 |
| Rural | 2012 | 21.484 |
| Rural | 2013 | 23.141 |
| Rural | 2014 | 22.32 |
| Rural | 2015 | 25.465 |
| Rural | 2016 | 24.425 |
| Rural | 2017 | 25.665 |
| Rural | 2018 | 24.833 |
| Rural | 2019 | 24.669 |
| Rural | 2020 | 26.552 |

**Supplementary Table 4.** Age-adjusted mortality rate (AAMR) stratified by urbanization for cardiovascular mortality and viral hepatitis in US adults from 1999 to 2020.

| **Hepatitis** | **Year** | **Age Adjusted Rate** | **Age Adjusted Rate Lower 95% Confidence Interval** | **Age Adjusted Rate Upper 95% Confidence Interval** | **Age Adjusted Rate Standard Error** |
| --- | --- | --- | --- | --- | --- |
| Hepatitis A | 1999 | 0.504 | 0.408 | 0.6 | 0.049 |
| Hepatitis A | 2000 | 0.472 | 0.381 | 0.563 | 0.046 |
| Hepatitis A | 2001 | 0.375 | 0.3 | 0.464 | 0.041 |
| Hepatitis A | 2002 | 0.407 | 0.327 | 0.501 | 0.043 |
| Hepatitis A | 2003 | 0.285 | 0.22 | 0.364 | 0.036 |
| Hepatitis A | 2004 | 0.266 | 0.204 | 0.34 | 0.033 |
| Hepatitis A | 2005 | 0.192 | 0.139 | 0.259 | 0.029 |
| Hepatitis A | 2006 | 0.174 | 0.126 | 0.235 | 0.027 |
| Hepatitis A | 2007 | 0.166 | 0.117 | 0.229 | 0.027 |
| Hepatitis A | 2008 | 0.154 | 0.109 | 0.211 | 0.025 |
| Hepatitis A | 2009 | 0.151 | 0.107 | 0.208 | 0.025 |
| Hepatitis A | 2010 | 0.121 | 0.082 | 0.172 | 0.022 |
| Hepatitis A | 2011 | 0.121 | 0.083 | 0.17 | 0.021 |
| Hepatitis A | 2012 | 0.128 | 0.088 | 0.18 | 0.022 |
| Hepatitis A | 2013 | 0.13 | 0.09 | 0.183 | 0.023 |
| Hepatitis A | 2014 | 0.114 | 0.079 | 0.161 | 0.02 |
| Hepatitis A | 2015 | 0.098 | 0.064 | 0.142 | 0.019 |
| Hepatitis A | 2016 | 0.099 | 0.067 | 0.141 | 0.018 |
| Hepatitis A | 2017 | 0.136 | 0.097 | 0.186 | 0.022 |
| Hepatitis A | 2018 | 0.215 | 0.164 | 0.278 | 0.028 |
| Hepatitis A | 2019 | 0.195 | 0.149 | 0.251 | 0.025 |
| Hepatitis A | 2020 | 0.219 | 0.171 | 0.277 | 0.026 |
| Hepatitis B | 1999 | 2.97 | 2.74 | 3.2 | 0.117 |
| Hepatitis B | 2000 | 2.768 | 2.547 | 2.989 | 0.113 |
| Hepatitis B | 2001 | 2.723 | 2.507 | 2.94 | 0.11 |
| Hepatitis B | 2002 | 2.805 | 2.587 | 3.022 | 0.111 |
| Hepatitis B | 2003 | 2.751 | 2.537 | 2.964 | 0.109 |
| Hepatitis B | 2004 | 2.616 | 2.41 | 2.822 | 0.105 |
| Hepatitis B | 2005 | 2.65 | 2.444 | 2.856 | 0.105 |
| Hepatitis B | 2006 | 2.593 | 2.391 | 2.796 | 0.103 |
| Hepatitis B | 2007 | 2.365 | 2.175 | 2.556 | 0.097 |
| Hepatitis B | 2008 | 2.658 | 2.457 | 2.858 | 0.102 |
| Hepatitis B | 2009 | 2.274 | 2.09 | 2.458 | 0.094 |
| Hepatitis B | 2010 | 2.372 | 2.188 | 2.556 | 0.094 |
| Hepatitis B | 2011 | 2.274 | 2.092 | 2.455 | 0.092 |
| Hepatitis B | 2012 | 2.27 | 2.091 | 2.449 | 0.091 |
| Hepatitis B | 2013 | 2.299 | 2.121 | 2.478 | 0.091 |
| Hepatitis B | 2014 | 2.448 | 2.266 | 2.63 | 0.093 |
| Hepatitis B | 2015 | 2.166 | 1.995 | 2.338 | 0.087 |
| Hepatitis B | 2016 | 2.19 | 2.018 | 2.363 | 0.088 |
| Hepatitis B | 2017 | 2.195 | 2.025 | 2.364 | 0.087 |
| Hepatitis B | 2018 | 2.209 | 2.041 | 2.377 | 0.086 |
| Hepatitis B | 2019 | 2.312 | 2.139 | 2.485 | 0.088 |
| Hepatitis B | 2020 | 2.555 | 2.377 | 2.734 | 0.091 |
| Hepatitis C | 1999 | 12.34 | 11.871 | 12.809 | 0.239 |
| Hepatitis C | 2000 | 13.444 | 12.958 | 13.93 | 0.248 |
| Hepatitis C | 2001 | 14.932 | 14.426 | 15.438 | 0.258 |
| Hepatitis C | 2002 | 16.292 | 15.768 | 16.816 | 0.267 |
| Hepatitis C | 2003 | 16.781 | 16.255 | 17.308 | 0.269 |
| Hepatitis C | 2004 | 17.461 | 16.93 | 17.992 | 0.271 |
| Hepatitis C | 2005 | 17.784 | 17.254 | 18.315 | 0.271 |
| Hepatitis C | 2006 | 19.969 | 19.414 | 20.524 | 0.283 |
| Hepatitis C | 2007 | 20.913 | 20.352 | 21.473 | 0.286 |
| Hepatitis C | 2008 | 21.265 | 20.707 | 21.823 | 0.285 |
| Hepatitis C | 2009 | 21.438 | 20.884 | 21.991 | 0.282 |
| Hepatitis C | 2010 | 21.277 | 20.73 | 21.824 | 0.279 |
| Hepatitis C | 2011 | 22.431 | 21.877 | 22.984 | 0.283 |
| Hepatitis C | 2012 | 23.585 | 23.022 | 24.148 | 0.287 |
| Hepatitis C | 2013 | 23.809 | 23.25 | 24.369 | 0.285 |
| Hepatitis C | 2014 | 24.124 | 23.566 | 24.682 | 0.285 |
| Hepatitis C | 2015 | 24.33 | 23.775 | 24.886 | 0.283 |
| Hepatitis C | 2016 | 23.661 | 23.117 | 24.204 | 0.277 |
| Hepatitis C | 2017 | 22.976 | 22.446 | 23.506 | 0.271 |
| Hepatitis C | 2018 | 21.413 | 20.904 | 21.922 | 0.26 |
| Hepatitis C | 2019 | 19.384 | 18.904 | 19.864 | 0.245 |
| Hepatitis C | 2020 | 21.557 | 21.054 | 22.061 | 0.257 |

**Supplementary Table 5.** Age-adjusted mortality rate (AAMR) stratified by viral hepatitis serotype for cardiovascular mortality and viral hepatitis in US adults from 1999 to 2020.


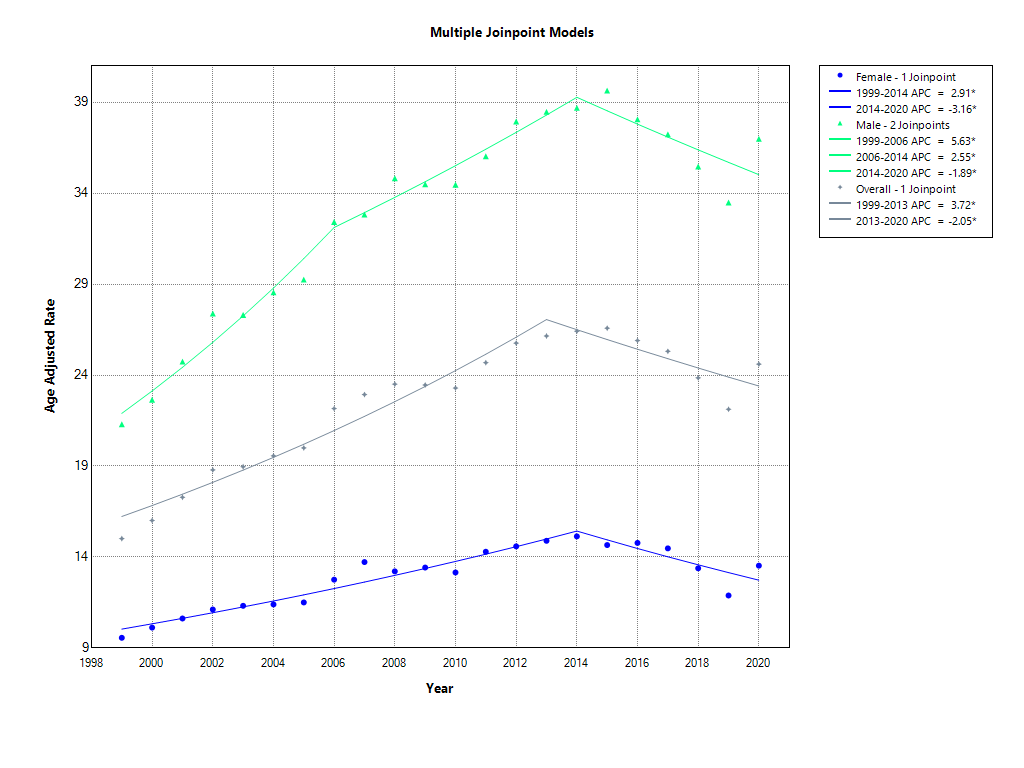


**Supplementary Figure 1.** Joinpoint regression trends of gender for cardiovascular mortality and viral hepatitis in US adults from 1999 to 2020.


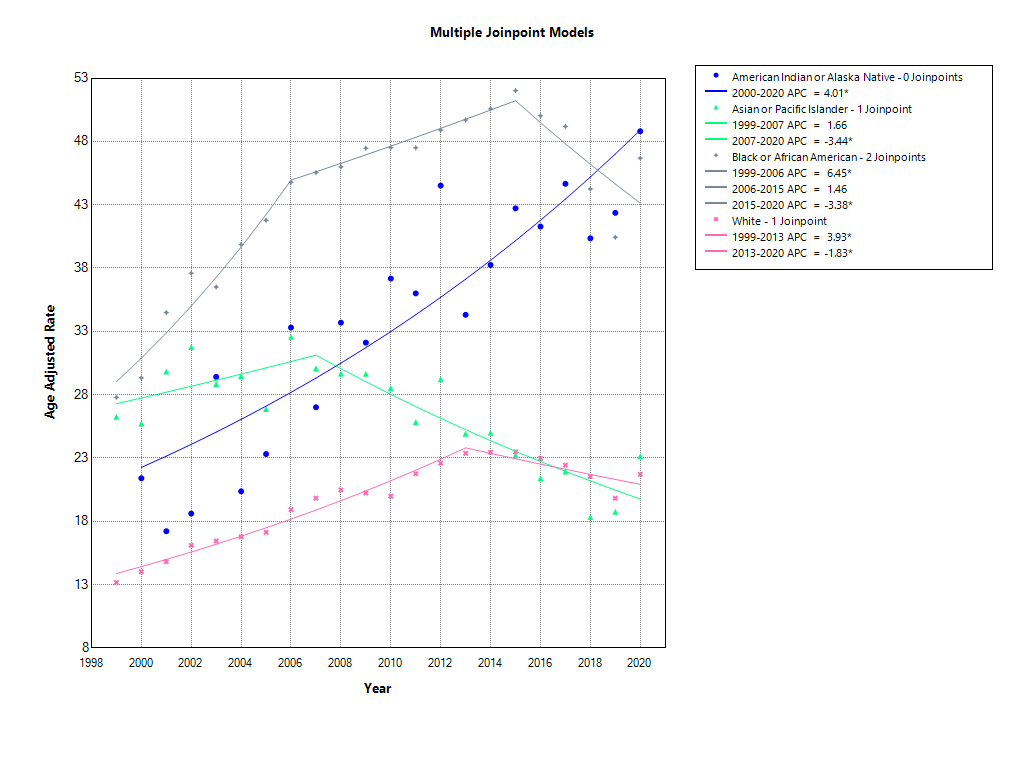


**Supplementary Figure 2.** Joinpoint regression trends of race for cardiovascular mortality and viral hepatitis in US adults from 1999 to 2020.


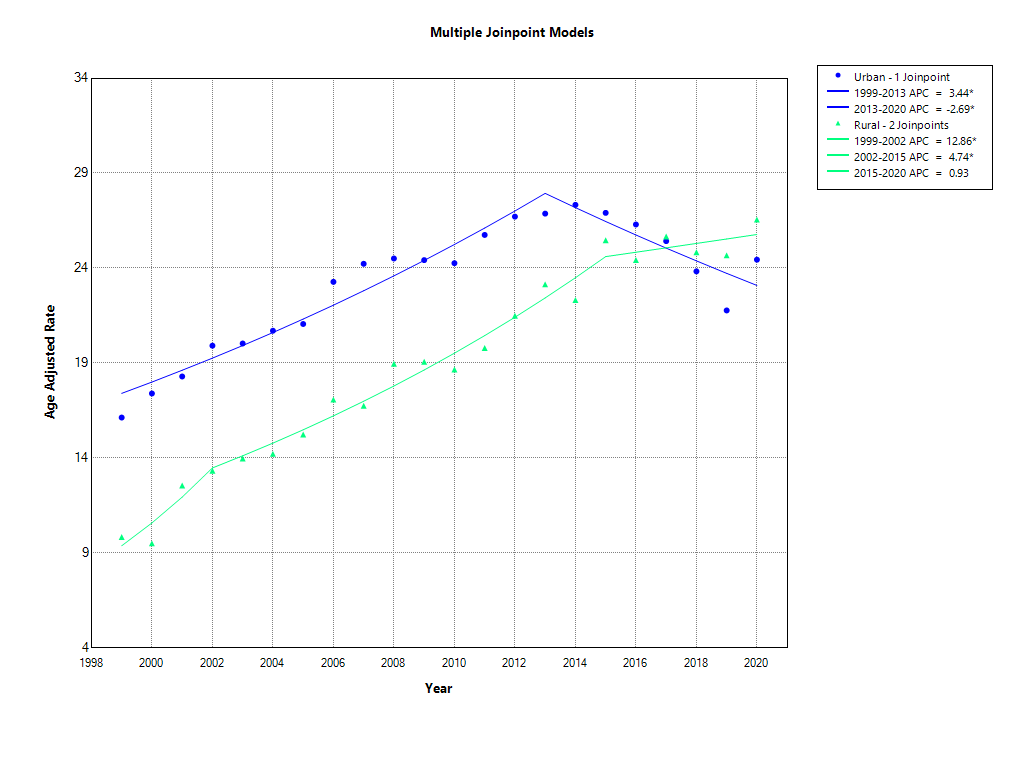


**Supplementary Figure 3.** Joinpoint regression trends of urbanization for cardiovascular mortality and viral hepatitis in US adults from 1999 to 2020.


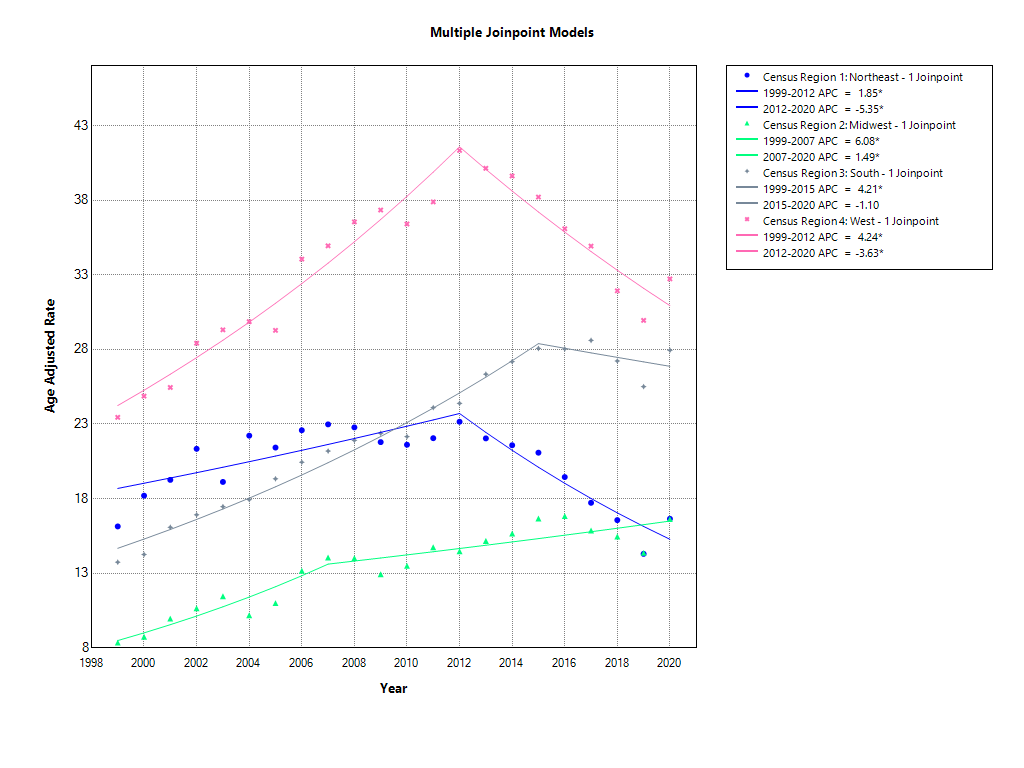


**Supplementary Figure 4.** Joinpoint regression trends of US census regions for cardiovascular mortality and viral hepatitis from 1999 to 2020.


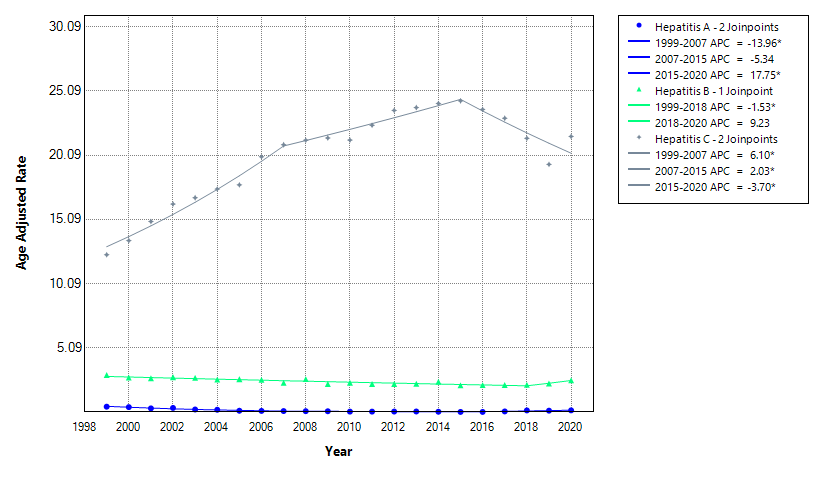


**Supplementary Figure 5.** Joinpoint regression trends by viral hepatitis serotype for cardiovascular mortality and viral hepatitis in US adults from 1999 to 2020.
